# Memory influences haptic perception of softness

**DOI:** 10.1038/s41598-019-50835-4

**Published:** 2019-10-07

**Authors:** Anna Metzger, Knut Drewing

**Affiliations:** Justus-Liebig-University Giessen, Department of General Psychology, Giessen, Otto-Behaghel-Strasse 10F, D-35394 Giessen, Germany

**Keywords:** Perception, Human behaviour

## Abstract

The memory of an object’s property (e.g. its typical colour) can affect its visual perception. We investigated whether memory of the softness of every-day objects influences their haptic perception. We produced bipartite silicone rubber stimuli: one half of the stimuli was covered with a layer of an object (sponge, wood, tennis ball, foam ball); the other half was uncovered silicone. Participants were not aware of the partition. They first used their bare finger to stroke laterally over the covering layer to recognize the well-known object and then indented the other half of the stimulus with a probe to compare its softness to that of an uncovered silicone stimulus. Across four experiments with different methods we showed that silicon stimuli covered with a layer of rather hard objects (tennis ball and wood) were perceived harder than the same silicon stimuli when being covered with a layer of rather soft objects (sponge and foam ball), indicating that haptic perception of softness is affected by memory.

## Introduction

Our perception of the environment is not solely determined by its physical properties but can be partially modulated top-down. Such top-down influences are multiple and of various kinds, for instance we can voluntary change the sensory input by moving our sensors to relevant object locations^1^ or move them optimally to extract relevant object properties^2^, deploy our attention to relevant locations^3^ or features of an object^4^ or disambiguate an ambiguous figure by expecting a certain object (for a review on top-down influences see Gilbert & Li, 2013^5^). Further on, perception can be modulated by higher-level cognitive states, such as memory or motivation^6–15^. For instance, typical colours of objects influence the visual perception of their colour^12,14,15^. When participants were asked to adjust the colour of a well-known object so that it appeared gray, their matches were slightly shifted towards the colour that is opponent to the object’s typical colour. For instance, a banana appeared gray to participants when it was adjusted to be slightly bluish, which is the opponent colour of the banana’s typical yellow colour^12^. However, when participants adjusted the colour of gray objects (like a golf ball or other objects which do not have a typical colour, like striped socks), the matches did not differ from neutral gray^14,16^. These effects demonstrate that stimuli with the same physical colour properties are perceived differently depending on prior knowledge of their typical colour.

Such influence of typical, remembered colours of objects on the perception of their colours could be successfully predicted assuming Bayesian integration of information^17^, a model of optimal combination of evidences from multiple sources^18,19^. The Bayesian framework of perception assumes that when individuals perceive a certain quality of the environment, they combine their beliefs about the world (prior knowledge, modelled as the probability density function of the states of the world) with their sensory evidence (likelihood of sensory data given the world state) to infer the probability of the world state given the data (posterior). The combination of the two sources of evidence corresponds to a multiplication of two distributions (prior and likelihood distributions), which results in another posterior probability distribution. If assuming that prior and likelihood distributions are Gaussian, then the posterior is also Gaussian, with smaller variance and the mean being the weighted average of the two initial distributions (with weights being proportional to the inverse variances). In other words, combining the two sources of evidence has the advantage of obtaining a more precise estimate, which corresponds to a hypothetically optimal performance.

In haptic perception, influences of prior knowledge have been reported for heaviness perception: A small object feels heavier than a large one with the same weight (size-weight illusion) and an object made of polystyrene is perceived heavier than an object with the same weight but made of lead (material-weight illusion)^20–24^. Objects made of lead usually weigh more than polystyrene objects, and small objects usually weigh less than large ones. The heaviness effects have often been explained by a mismatch between expected and actual heaviness^25–28^. According to the mismatch hypothesis, a small object is perceived to be heavier than it actually is because it had been expected to weigh less. However, that perception is shifted away from expectations rather than towards them is at odds with observations in other corresponding cases (e.g. colour or skin tone perception)^11–14^. Other authors hence link the shift in weight perception to an effect of assumptions on the object’s density, rather than on weight. They suggest that people expect denser objects to be heavier than less dense objects. As a consequence, smaller, denser objects should feel heavier than larger, less dense objects of the same mass, explaining the “size-weight” illusion by a perceptual shift towards expectation^29–31^. A recent study on the influence of weight expectations for every-day objects suggested a perceptual shift away from the object’s typical weight in a first experiment, but towards it in the second experiment^32^. These findings demonstrate that the mechanisms of how expectations and prior assumptions actually influence perceived heaviness are far from being understood.

In the present study we aim to identify how the knowledge of an object’s typical softness influences haptic perception of this object’s softness. Softness is the subjective experience of an object’s ability to deform under pressure^33^. Its physical correlate is compliance (measured in mm/N). In contrast to the weight of an object (which is an extensive physical property), softness is an intensive object property, i.e. the weight of the object changes with its size (density expressing the interdependence), whereas its softness remains the same. Thus, when building up knowledge about the weight of objects, both their size and density have to be taken into account, making it difficult to figure out unequivocally which learned associations affect weight perception. In contrast, for an intensive object property like softness, built up knowledge likely relates exclusively to softness so that a direct link can be made between prior knowledge about softness and its perception, allowing us to generalize the effect of prior knowledge on perception from visual^12,14,15^ to haptic perception.

**Figure 1:**
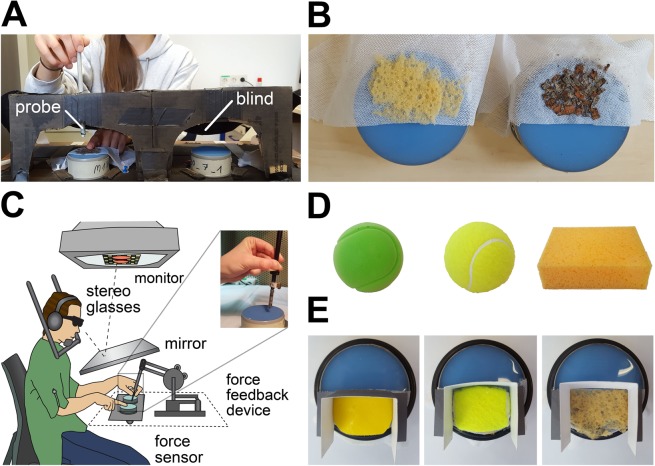
Setup and stimuli. **(A)** Setup of Experiments 1, 2 and 4. Participants sat in front of the experimenter. They made lateral strokes with the index finger to explore the surfaces of the stimuli and indented them then with a probe. A cardboard box with a blind prevented participants from seeing the stimuli. **(B)** Stimuli Experiment 1. Silicone rubber disks were partly covered with thin layers of bark (wood condition) or sponge glued onto elastic cloth. **(C)** Setup Experiment 3. Participants sat at a visuo-haptic workbench. Exploration of the stimuli was as in Experiments 1, 2 and 4. The probe was connected to a PHANToM force feedback device (for details about this setup see^41^). **(D)** Tennis ball and sponge used for the covers in Experiments 2 and 4 and foam ball used for the cover in Experiment 3. **(E)** Stimuli in Experiments 2–4. Silicone rubber stimuli were covered with pieces of foam, or a tennis ball, or a slice of a sponge in Experiment 3. The foam ball cover was not used in Experiments 2 and 4.

In order to judge an object’s softness, people usually repeatedly indent their bare finger or a tool into the object, or squeeze it between their fingers^2,34,35^. These movements generate several sensory cues to softness (such as the ratio between force and displacement, the deformation of the surface, and vibration of the surface caused by touch)^36–39^. To assess the influence of prior knowledge on softness perception, we aimed to elicit the memory of objects before the inspection of their softness (via object indentations). For this purpose we chose objects, which had 1. Different typical surfaces that can be recognized by a lateral stroke and 2. Different typical compliances (sponge and wood in Experiment 1; sponge and tennis ball in Experiment 2 and 4; foam ball, sponge and tennis ball in Experiment 3). We produced silicone rubber stimuli with varying softness and thin slices of the objects, which were partially laid over the silicone rubber stimuli (Fig. 1B,E). Participants first stroke laterally (with the index finger of one hand, without indenting) over the half of the stimulus covered with the object’s surface. Then, they indented the uncovered half of the stimulus with a probe which they held in the other hand in order to judge softness. Participants could not look at the stimuli or their hands during haptic inspection, and were not aware of the bipartite design of the stimuli (Fig. 1A,C). In different conditions, participants were actually inspecting the same silicone stimuli, which were covered with different object surfaces. This way the object’s compliance and thus the perceptual cues to softness were kept constant between conditions, so that differences in perceived softness could be lead back to the object surface first explored and thus to its remembered softness. We performed in total 4 experiments: In all experiments participants were asked on every trial to compare two stimuli and decide which one felt softer. In order to measure perceived softness, we assessed Points of Subjective Equality (PSEs) of one standard, compared to a set of comparison stimuli.

In Experiment 1, we used a sponge as a soft object and wood as a hard one. In this experiment, object surfaces were laid over the comparison stimuli, whereas the standard stimulus was uncovered. We also included a baseline condition in which both stimuli - standard and comparison - were uncovered silicon. Participants performed a two interval forced choice task by which we measured perceived softness: On each trial they compared the standard with one of the comparison stimuli and decided which one felt softer. Perceived softness was estimated as the point in which the comparison and the standard were indistinguishable. In this experiment we did not find a significant difference between the wood and the baseline condition. We suspected that the wood surface crafted by us (Fig. 1B) failed to be recognized as wood or the difference between the remembered compliance of the wood and the felt compliance of the silicon was to large, rendering the bipartite stimulus incredible. Thus we repeated this experiment (Experiment 2) using as a hard object a tennis ball, which was reliably recognized by a lateral stroke with a finger. Additionally we explicitly told participants which objects were involved in the experiment, blocked the conditions in order to decrease the interference between the remembered softness of the two objects, performed 10 training trials in which participants were given feedback on whether they correctly recognized the objects by a lateral stroke and included a questionnaire to make sure participants did recognize the objects and did not notice the manipulation. In Experiment 3 we aimed to replicate the results of Experiment 1 and 2 with different methods, in order to decrease the probability that the observed effects were methodological artefacts. In this experiment we used a tennis ball as a hard object and a foam ball and sponge as soft objects. To measure perceived softness, we used a matching task: On each trial, participants actively selected a comparison stimulus that matched the perceived softness of the standard stimulus. They initially received one of the two hardest or softest comparison stimuli in the set and could exchange it (by advising the experimenter to provide softer or harder stimuli) until they had the feeling that the softness of the comparison and the standard was equal. In this experiment, the standard stimulus was covered with object surfaces and the comparison stimuli were left uncovered. Besides these modifications the procedure was kept as in Experiment 1 and 2. In a last experiment (Experiment 4) we tested a hypothesis derived from the Bayesian framework. Assuming that prior knowledge is integrated optimally with available sensory information it can be expected that the influence of prior knowledge on perception increases as uncertainty of the sensory information increases^40^. To test this hypothesis we designed Experiment 4, which contained two conditions: in the first condition participants were allowed to indent the stimuli only once and in the second as often as they wanted. It was shown that in haptic softness perception discrimination thresholds decrease with increasing number of indentations^41^. We expected that with unlimited exploration of the stimuli participants would gain a more precise estimate of the object’s softness as compared to the case in which they have to base their softness estimate on a single indentation. Thus we expected a smaller effect of prior knowledge on softness perception with an unlimited exploration. In this experiment we used the sponge and the tennis ball as objects and laid the object surfaces over the standard stimuli. Here, we used the 2IFC task combined with the method of constant stimuli to measure perceived softness. Additionally we estimated from the responses of participants just noticeable differences (JND) as a measure of perceptual precision.

In all experiments we expected that perceived softness would be biased towards the memorized softness that is associated with the surface of the stimulus. In all experiments, we assess perceived softness of the standard stimulus. In Experiment 3 and 4, the standard stimuli were covered with surfaces of known objects, and we expected their perceived softness to be biased towards the object’s typical softness. In contrast, in Experiment 1 and 2, it were the comparison stimuli that were covered with object surfaces, and we correspondingly expected the standard’s softness to be biased away from the known object’s softness. In all experiments but Experiment 1 participants were explicitly told before the experiment which objects were involved in the experiment.

## Results

For all experiments we estimated perceived softness for all conditions. In Experiment 1 we used an adaptive method, in which the compliance of the comparison stimuli is increased/decreased until participants changed their judgment from softer to harder or vice versa. Perceived softness can then be estimated as the average compliance of comparisons at which such “reversals” occurred; In Experiment 2 & 4 we used the method of constant stimuli in which the standard is paired with each comparison a constant number of times. The responses of the participants constitute the psychometric function to which we fitted cumulative Gaussian functions and estimated the perceived softness of the standard as the 50% discrimination threshold. In Experiment 3 where we used a matching method perceived softness was estimated as the average over comparisons which participants matched to the standard.

**Figure 2:**
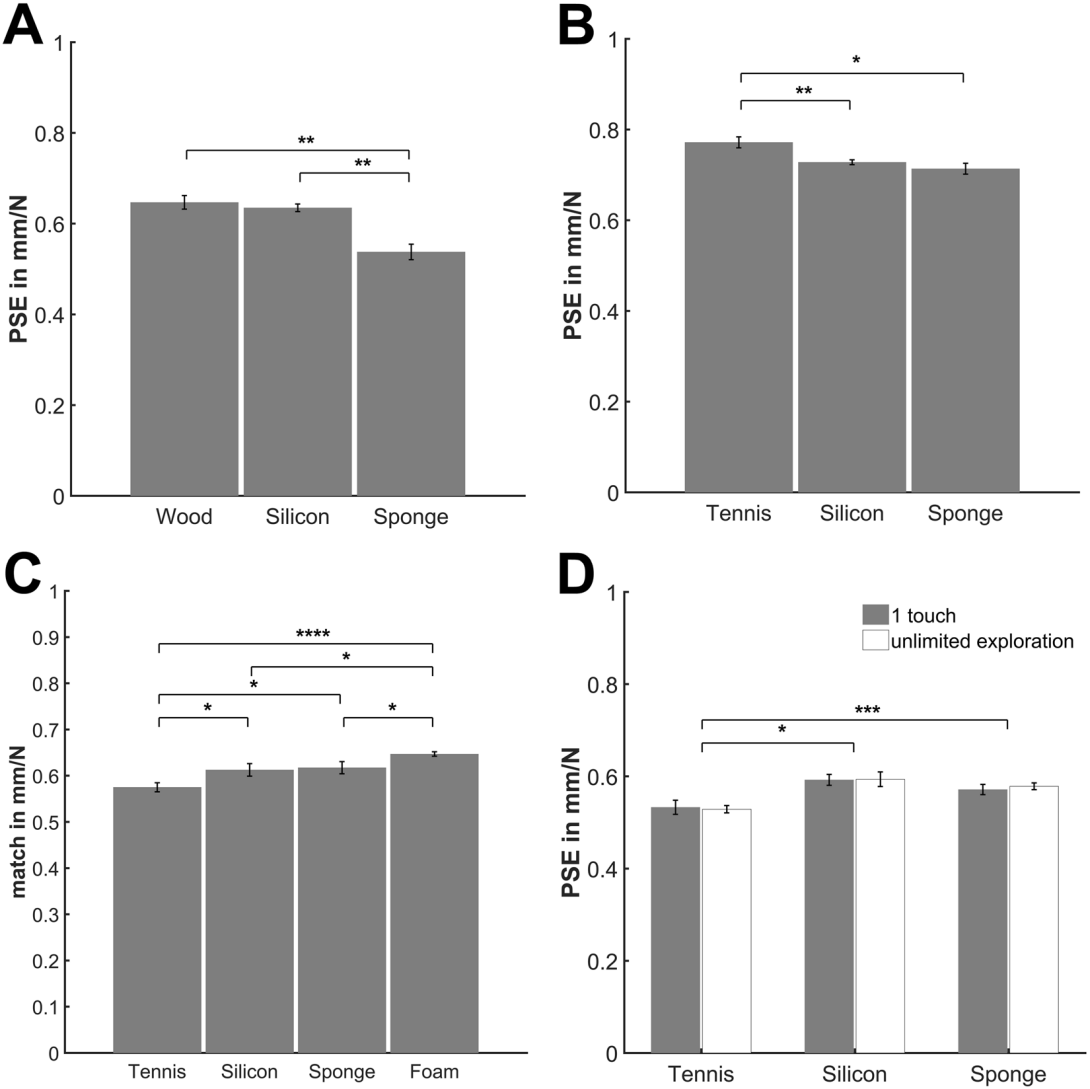
Average PSEs and matches. (**A**) Experiment 1: Average perceived softness of an uncovered silicon standard measured with comparison stimuli covered with a layer of wood, sponge or uncovered. It is predicted that the standard will be perceived equal to harder comparisons when they are covered with the sponge and to softer comparisons when they are covered with the wood as compared to when they are uncovered. (**B**) Experiment 2: Average perceived softness of an uncovered silicon standard measured with comparison stimuli covered with a layer of a tennis ball, sponge or uncovered. It is predicted that the standard will be perceived equal to harder comparisons when they are covered with the sponge and to softer comparisons when they are covered with the tennis ball as compared to when they are uncovered. (**C**) Experiment 3: Average matches of a silicon rubber standard with a foam ball, a sponge, a tennis-ball cover or no cover to uncovered silicon rubber comparisons. It is predicted that the standard will be perceived harder when covered with the tennis ball and softer when covered with the sponge or foam ball as compared to when being uncovered. **(D)** Experiment 4: Average perceived softness of a silicon stimulus covered with a layer of a tennis ball, sponge or uncovered measured with uncovered silicon comparisons and differently long explorations of the stimuli (one touch or unlimited). It is predicted that the standard will be perceived harder when covered with the tennis ball and softer when covered with the sponge as compared to when being uncovered with effects being smaller with a long exploration. Error bars represent the within-subject standard error^60^. *p < 0.05; **p < 0.005; ***p < 0.0005; ****p < 0.00005.

The results are plotted in Fig. 2. The known softness of the apparent objects influenced perceived softness of silicone rubber stimuli, independent of the used methods (Experiment 1: *F*(2,18) = 12.35, *p* < 0.001; Experiment 2: *F*(2,34) = 5.72, *p* = 0.007; Experiment 3: *F*(3,39) = 5.52, *p* = 0.003; Experiment 4: *F*(2,18) = 5.97, *p* = 0.01, ANOVAs on PSEs or matches with the within-subject factor *object surface*: Experiment 1 [wood, silicon, sponge], Experiment 2 [tennis ball, silicon, sponge], Experiment 3 [foam ball, sponge, silicon, tennis ball]). We further tested our directional hypothesis on the influences of objects’ known softness by one-sided *t*-tests. We found the predicted effects across different methods in all our experiments. In Experiment 1 comparison stimuli covered with a sponge layer were perceived to be softer than the ones covered with bark so that the standard was perceived to be equal to a harder sponge comparison than a bark comparison, *t*(9) = 3.53, *p* = 0.003). Similarly, in Experiment 2 comparison stimuli covered with a layer of a tennis ball were perceived to be harder than the same stimuli covered with layers of the sponge, so that the standard was perceived equal to a harder sponge comparison than a tennis ball comparison, *t*(17) = 2.46, *p* = 0.012. Also as predicted, in Experiment 3 participants matched the standard silicon stimulus covered with an object surface to harder comparison stimuli when they were made to believe that they explored a tennis ball as compared to the case they thought to explore a foam ball (*t* (13) = −6.11, *p* < 0.001) or a sponge (*t* (13) = −2.15, *p* = 0.0254). And finally, also in Experiment 4, the standard silicon stimulus covered with a layer of a tennis ball was perceived equal to harder comparison stimuli than when it was covered with a layer of a sponge (*t* t(9) = −4.89, *p* < 0.001, PSEs averaged over the two *exploration length* conditions). We did not always find perceptual differences between conditions in which the silicon stimuli were covered with layers of well-known objects as compared to the baseline condition in which the silicon stimuli were uncovered (Fig. 2, Exp. 1, bark vs. silicon: *t*(9) = 0.69, *p* = 0.253; Exp. 2, sponge vs. silicon: *t*(17) = 1.01, *p* = 0.157; Exp. 3, sponge vs. silicon: t(13) = −0.19, p = 0.424; Exp. 4, sponge vs. silicon: *t*(9) = 0.78, *p* = 0.228). However, the direction of the PSE shift as compared to the baseline in these cases was always as predicted besides in Experiment 4.

**Figure 3:**
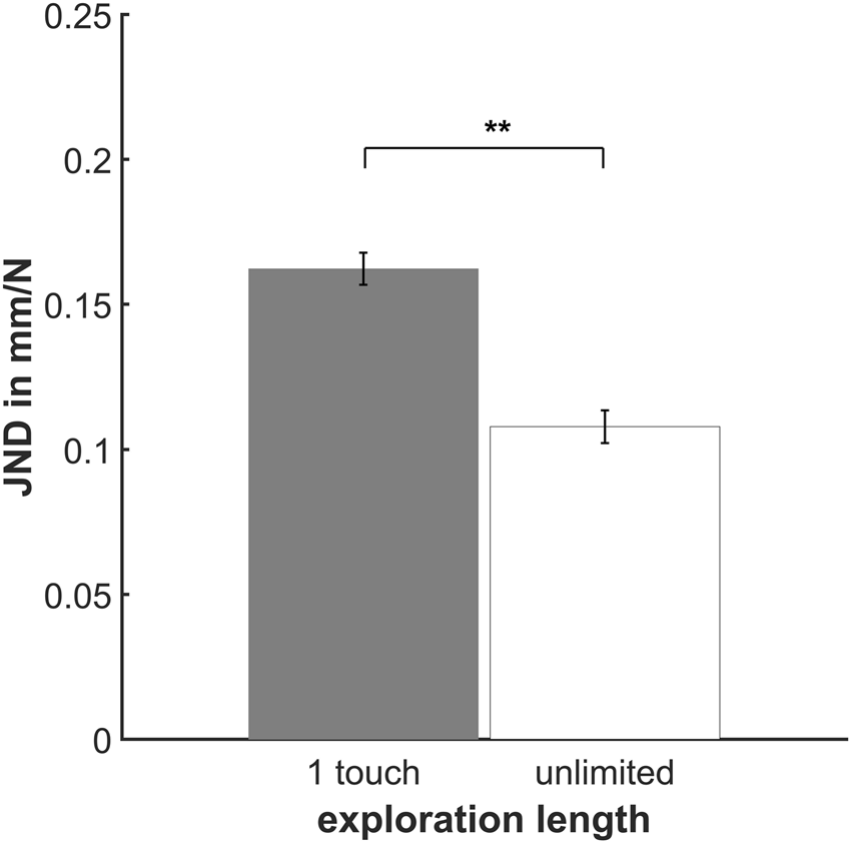
Average JNDs. Experiment 4: Average discrimination thresholds for softness of silicon stimuli explored with a pen given different length of the exploration (one touch or unlimited). JNDs were averaged over the *object surface* conditions. Error bars represent the within-subject standard error^60^. **p < 0.005.

In Experiment 4 we tested whether the influence of prior knowledge decreases when precision of sensory information increases. Discrimination thresholds were significantly modulated by the manipulation of the exploration length (one touch vs. unlimited exploration, *F*(1,9) = 24.08, *p* = 0.001) and almost halved when participants were allowed to explore the stimuli as much as they wanted (Fig. 3), indicating that the precision of the sensory information increased. However, perceptual differences between different *object surface* conditions were almost identical with different exploration lengths of the stimuli (Fig. 2D, *F*(1,9) = 0.03, *p* = 0.871). There was no significant effect of *object surface* on the JNDs (*F* (2,18) = 0.463, *p* = 0.637).

After completion of Experiment 2–4, participants reported in the questionnaire if they noticed that the stimuli were manipulated (i.e. not homogeneous, intact objects but object surfaces laid over silicon stimuli). Only data of participants who did not report to have noticed such a manipulation was analyzed. Participants also reported their guess on the purpose of the study in the questionnaire. 81% of participants had no idea about the purpose of the study, too general guesses (e.g. softness perception) or guessed wrong (e.g. effect of presentation order). Remaining participants guessed that we were investigating the influences of texture properties (e.g. fluffy or smooth) on softness perception. This is in principle correct, but too general to be problematic, as they did not mention memory of familiarity and presumably focused on the different properties of objects’ surfaces. Importantly, results did not change significantly, when these participants were removed from analyses.

## Discussion

In four experiments, using different methods, we repeatedly replicated the main effect of object surface: silicon stimuli which were believed to be hard every-day objects felt harder than the same stimuli believed to be soft every-day objects. When comparing perceived softness in the conditions with object surfaces to the baseline, we found significant perceptual shifts for soft as well as for hard objects. For most objects (8 out of 9 cases) the measured perceptual shifts were in the predicted direction. In some cases this difference did not reach significance, but the order of objects was always as predicted, i.e. the memory of a hard object made the silicon always feel harder as compared to the case when a soft object was remembered. The failure to show a difference from the baseline might be due to object idiosyncrasies, i.e. as for color vision, memory effects massively vary between different objects^14^ or due to the perception of the silicon itself, e.g. assignment of a typical softness during the experiment relative to the objects used in this experiment. We thus can conclude that the memory of typical object softness influences perceived softness towards expectation. In contrast to weight, where it is yet not clear in which direction expectations contribute to perception, for softness a direct link can be made between expected and perceived softness, because it is an intensive physical property.

The shifts in perceived softness induced by the memory of objects’ typical softness varied between 0.005–0.1 mm/N. This corresponds to a relative shift of 5–10% as compared to the difference in perceived softness of the silicon standard and the measured compliances of the objects. Thus, although memory significantly affects softness, its effect is rather limited. This is expected based on previous research on color: Hansen and colleagues^12^ estimated the effect size of memory color as around 8% relative to the typical color of the stimuli. In fact, although the perceptual systems may make use of prior information to reduce perceptual uncertainty, perception must strongly depend on the physical world in order to be useful.

Our results are only partially in agreement with the Bayesian model of perception. We find perceptual shifts towards the known softness of the objects consistent with the idea that prior knowledge is integrated with sensory information. However, the prediction that the influence of prior knowledge would decrease if perceptual precision increases, did not hold. Specifically, in Experiment 4 in one condition participants were allowed to indent the silicone stimuli only once and in the second as often as they wanted. As expected, multiple indentations increased precision. In both conditions we found memory effects on softness, however, these effects were not different between the two conditions. This is at odds with optimal integration, because lower precision should correspond to higher influence of the memory prior. Deviations from optimality have been previously reported (see for a review: Rahnev & Denison, 2016^42^). Because optimality is often the hypothesized outcome, findings of suboptimality may be underreported in the literature^43^. Our findings increase evidence that optimal integration in perception may not be the rule.

There is growing evidence that perception is affected by higher-level cognitive states, like the desirability of objects^6^, action capabilities^7–9^, arousal^10^, and categorical knowledge^11–15^. However, whether perception actually is^44,45^ or is not^46–48^ penetrable by cognition is presently a hotly debated topic. There is strong evidence from theoretical, behavioral, neurophysiological and clinical studies strongly supporting the predictive coding framework of perception, in which top-down pathways send information based on prior experiences which is then combined with incoming sensory information^44,45,49,50^. For instance, imagined and real natural sounds (e.g. traffic noise or birds singing) in absence of any visual input could be decoded in the early visual cortex^51^. Also, several perceptual phenomena are difficult to explain without assuming a penetration of perception by cognition. For instance seeing a Dalmatine dog in an impoverished black and white image after knowing that there is a dog or the striking individual differences in the perception of “the dress” (white-gold to some people and blue-black to others), which can most reasonably be explained by individually different assumptions about the illumination^52–54^. Here, effects are so strong that there cannot be any doubt that these are perceptual experiences rather than effects from cognitive judgements. Nevertheless, there is some doubt about behavioral evidence for the penetrability of perception by cognition. For instance, Firestone and Scholl^46^ argue that evidence is missing because existing studies are actually targeting either a different influence e.g. attention or do not measure effects on perception but e.g. on (cognitive) judgements. While it is arguable that influences of attention cannot be interpreted as cognitive influences on perception^45^ and we anyways are confident about actually targeting the influence of prior knowledge and not attention, we cannot proof that measured effects are purely perceptual. We aimed to minimize potential cognitive influences by using (in all experiments) methods that focus on basic perceptual decisions, instead of asking for more abstract responses. Nevertheless, because in this study we measured appearance and participants could differentiate between conditions, every perceptual shift might be also confounded with a decisional bias^55^. However, assuming that perception is penetrable by cognition it is necessary to assume that there is no distinct division between observation and inference. However, there is evidence that influences of prior knowledge on visual perception are more of perceptual and less cognitive nature. For the memory color effect it was shown that when recording brain responses to well-known color-diagnostic objects in absence of chromatic signals, activity in the early visual cortex (V1) allows predicting the memory color of these objects^56^. Results are in line with the predictive coding theory:^50^ higher visual areas send predictions of expected object colors to V1, where they are compared to incoming sensorial information. This evidence supports the idea that a similar mechanism applies also for the influence of prior knowledge on haptic perception.

## Methods

### Participants

Ten volunteers (7 female, all right-handed, average age 23.8 years) participated in Experiment 1. In Experiment 2 the final sample consisted of 18 participants (14 female, all right-handed, average age 26.1 years). Four participants were excluded because they reported in the debriefing that they had noticed the manipulation of the stimuli. In Experiment 3 the final sample comprised 14 participants (9 female, 1 left-handed, average age 26.8 years). Data of two participants had to be excluded from analyses, because they noticed the manipulation of the stimuli. The total sample in Experiment 4 consisted of 10 participants (5 female, all right handed, average age 24.8 years). Two participants had to be excluded because they noticed the manipulation of the stimuli. None of the participants participated in more than in one of the experiments. In all experiments none of the participants reported sensory or motor impairments of the hands and they were reimbursed for their time. The study was approved by the local ethics committee at Justus-Liebig University Giessen LEK FB06 (SFB-TPA5, 22/08/13) and was in line with the declaration of Helsinki from 2008. Written informed consent was obtained from each participant.

### Stimuli

We produced softness stimuli with different compliances by mixing a two-component silicon rubber solution (AlpaSil EH 10:1) with different amounts of silicon oil (polydimethylsiloxane, viscosity 50 mPa/s) and pouring it into cylindrical plastic dishes (75 mm diameter × 38 mm high). The final set comprised 12 stimuli: 1 standard and 11 comparison stimuli. In Experiment 1 and 3 the standard had the compliance of 0.67 mm/N and the comparison stimuli had compliances of 0.18, 0.27, 0.37, 0.48, 0.57, 0.67, 0.77, 0.88, 0.97, 1.09, 1.16 mm/N. For Experiment 2 and 4 which were performed after 1 and 3, we produced new sets of stimuli, because the stimuli were easily partially destroyed by repeated indentations with the pen in the former two experiments. In the new set for Experiment 2 the standard had a compliance of 0.68 mm/N and comparisons had compliances of 0.18, 0.28, 0.38, 0.48, 0.59, 0.68, 0.78, 0.88, 0.99, 1.09 and 1.19 mm/N. In the new set for Experiment 4 the standard had a compliance of 0.54 mm/N and comparisons had compliances of 0.14, 0.24, 0.37, 0.38, 0.48, 0.53, 0.65, 0.68, 0.75, 0.85 and 1.02 mm/N. The compliance of the stimuli was measured as described in^57^. The compliance of the standards was chosen to be in between the compliances of the soft and the hard objects (tennis ball ~= 0.171 mm/N, sponge ~= 1.59 mm/N, foam ball ~ = 2.14 mm/N).

We further produced different covers for the stimuli: wood, sponge, foam ball, tennis ball. For the wood and sponge stimuli (Fig. 1B) we cut thin slices of a sponge (Fig. 1D) and of the bark of a Norway maple (*Acer platanoides*) respectively, with a razor blade. The slices of sponge and bark were glued onto pieces of elastic cloth using spray glue (Fig. 1B). The covers could then be clamped over the softness stimuli and fixated on the floor of the cardboard box with drawing pins (Fig. 1A), so that they did not move when participants were striking over them. In Experiment 2–4, we used the same cover for the sponge stimulus; for the foam and the tennis ball stimuli (Fig. 1E) we cut similar pieces of ~5 × 3 × 0.8 cm (length × width × height) of a tennis and a foam ball (Fig. 1D) and fixated them on elastic cloth pieces using double-sided tape. In Experiment 2, the covers were fixated with rubber seal rings (EPDM) on the softness stimuli and surrounded by cardboard pieces to prevent participants from touching the silicon rubber(Fig. 1E).

### Design

The design of Experiments 1–3 comprised one within-subject variable: *object surface* over which participants stroke laterally before indenting the silicon stimuli (Exp. 1: sponge, wood and silicon; Exp. 2: sponge, tennis ball and silicon; Exp. 3: sponge, tennis ball, foam ball and silicon). Experiment 4 comprised two within-subject variables: *object surface* (sponge, tennis ball and silicon) and *exploration length* of the stimuli (1 touch vs. unlimited exploration). Conditions were presented in random order in Experiments 1 and 3 and blocked in Experiments 2 and 4. The two different *exploration length* conditions in Experiment 4 were performed on two different sessions, with the *unlimited exploration* condition being always performed in the second session to maximize the chance that precision would be higher in this condition. The *object surface* conditions were blocked within each session. In Experiment 2 every *object surface* condition was presented in a separate block resulting in 3 blocks and in Experiment 4 there were two blocks: in one block all sponge trials and half of the silicon trials were presented and in the other block all tennis ball trials and the other half of the silicon trials were presented. The order of blocks in Experiments 2 and 4 was counterbalanced across participants according to a Latin square design. The dependent variable was the estimate of perceived softness (PSE) in all experiments and in Experiment 4 additionally the estimated discrimination threshold (JND). In Experiments 1–3 individual PSEs were entered in a one-way repeated measures ANOVA with the within-subject factor *object surface*. In Experiment 4 individual PSEs and JNDs were submitted to a two-way repeated measures ANOVA with within-subject factors *object surface* and *exploratrion length*. In case ANOVA effects were significant, we tested our directed hypotheses using selected one-tailed pairwise comparisons.

### Procedure and setup

Experiments 1, 2 and 4 were performed at a small table, on which a cardboard box was mounted (Fig. 1A). The experimenter and the participant sat on opposite sides of the table. The cardboard box was opened on the side of the experimenter. In every trial, the experimenter placed two softness stimuli (the standard and a comparison) inside the box and covered the comparison stimulus in Experiments 1 and 2 and the standard stimulus in Experiment 4 with a cover according to the experimental condition (wood, sponge or none). The stimuli, the cover, the standard’s position (left or right randomized) and which stimulus had to be explored first (left or right randomized), was indicated to the experimenter by a custom computer program (MATLAB). After receiving the instruction on which side to start, participants reached inside the box with their left hand. The box was also open at the side facing them, but covered with a black cloth (blind) to allow exploration of the stimuli and at the same time prevent direct view of the stimuli. Independent of the stimulus, participants first made a stroke laterally over the surface of the stimulus with the left index finger, and indented the stimulus then with a probe, which they held in the right hand. The probe was inserted into the box through a small hole at the top of the box approximately above the stimulus. Participants were instructed to strike over the surface and to indent the stimulus only once. Exploration of the surface was not restricted to promote its recognition and recall of the memory about the associated object while the exploration of softness was restricted to control the sensory input and to limit the duration of the experiment. They were explicitly told to lift the left index finger before indenting the stimulus with the probe, to prevent them from feeling with this finger the displacement of the stimulus’ surface caused by indenting it with the probe in the right hand. Expectations of audio signals from contact with the stimuli were minimized by using a pen with a rubber tip as a probe. After participants explored the second stimulus (in the same way as the first one), they reported verbally which stimulus felt softer (left or right). The experimenter entered the answer in the computer program. In Experiment 1 we used a 1-Up-1-Down staircase^58^, in which the comparison stimuli were trial by trial adaptively selected to approach the point in which the comparison and the standard were indistinguishable. There were four 1-Up-1-Down staircases for each of the three conditions, two starting with the hardest, and two with the softest comparison. On each trial of a staircase, the response of the participant determined the comparison of the next trial of this staircase (if the comparison was perceived harder than the standard, a softer comparison was provided and vice versa). When participants changed their judgment from softer to harder or vice versa a “reversal” was counted. The experiment consisted of blocks of 12 trials, including one trial from each of the 12 staircases. The order of trials in a block was randomized. Staircases terminated after 10 reversals, resulting in 40 reversals for every condition. In Experiments 2 and 4 instead of a staircase we used the method of constant stimuli to measure perceived softness. The standard was paired with each comparison 8 times in every condition resulting in 88 trials per condition. In Experiment 2 there were thus in total 264 trials, which were performed on the same day within on average 2.25 hours. Experiment 4 consisted in total of 528 trials which were split into two sessions with an average duration of 2.6 hours.

Experiment 3 was performed at a visuo-haptic work bench (Fig. 1C), consisting of a force-feedback device to measure the position of the probe, a mirror to prevent direct sight of the stimuli and the participants’ hands, a monitor to display a schematic representation of the stimuli and the probe, that was used to guide hand movement (viewed through 3D-glasses and the mirror, no visual information on compliance was provided), and headphones that displayed white noise to mask audio signals from the stimuli. In every trial, participants were initially presented with two stimuli: a standard and a comparison. A custom-made program (C++) told the experimenter which stimuli s/he had to present. There were four experimental blocks. In each block, each of the four experimental conditions (standard stimulus covered with tennis ball, foam ball, sponge, no cover) was tested in two trials (random order); the standard stimulus was initially paired once with one of the two softest comparisons (which one was randomly determined) and once with one of the two hardest comparisons. This way initial comparison stimuli were clearly distinguishable from the standard, but it was difficult for participants to count the available stimuli. Participants were instructed to always explore the standard stimulus (on the right side) as follows: They were expected to first strike laterally over the stimulus’ surface using their right index finger, then lift the right finger and afterwards indent the stimulus with a probe which they held in their left hand. The experimenter helped to guide the finger of the participant to the cover of the standard. The comparison stimulus was explored only with the probe. Participants were free to explore each of the two stimuli, and to switch between the stimuli as often as they wanted. However, whenever they wanted to indent the standard they were instructed to strike over the surface of the stimulus anew. Their task was to find a comparison stimulus that matched the standard stimulus in perceived softness. For this purpose, participants had to instruct the experimenter to exchange the actual comparison stimulus with a softer or a harder one until they felt that the two stimuli matched. Furthermore, participants were encouraged to reverse in each trial at least once with the instruction from softer to harder or vice versa to find the stimulus which matched best to the standard. If participants asked for a stimulus that was softer or harder than the softest/hardest comparison stimulus, the same softest/hardest stimulus was delivered again. The final match was recorded by the experimenter.

In the end of all experiments there was a debriefing in which participants were shown the stimuli and were asked whether the stimuli matched their experience of the stimuli during the experiment. Data of participants who noticed the manipulation was excluded from analysis. Additionally participants filled in a questionnaire consisting of three questions: “Which and how many objects did you touch during the experiment?”, “What do you think is the purpose of the study?” and “Did you apply a particular strategy to complete the task?” In Experiment 1 data from the questionnaire was not collected in written from due to human error.

### Data analyses

Perceived softness was estimated in Experiment 1 as the average over the comparisons at which reversals occurred in the staircases and in Experiment 3 as the average over the final matches. In Experiments 2 and 4 we calculated for each participant, each condition, and each comparison stimulus the percentage of trials in which it was perceived to be softer than the standard. Combined for all comparisons these values, composed individual psychometric data, to which we fitted cumulative Gaussian functions using the *psignifit 4* toolbox^59^. From the fitted psychometric functions, we estimated the PSE as the 50% discrimination threshold. In Experiment 4 we additionally estimated the JNDs as the 84% discrimination threshold.

## Supplementary information

Supplementary data
