# Supplementary material for: Memory influences haptic perception of softness
Source: Sci Rep. 2019 Oct 7;9:14383. doi: 10.1038/s41598-019-50835-4 (PMC6779751; doi:10.1038/s41598-019-50835-4)
Supplement: Supplementary file 1 — Supplementary data [file 41598_2019_50835_MOESM1_ESM.pdf]

Memory influences haptic perception of softness

Anna Metzger & **Knut Drewing**

- Supplementary Material -

Experiment 1

Subject Nr	Condition	Reversals in the staircase in mm/N									
	wood	0.672	0.5745	0.672	0.5745	0.672	0.5745	0.9733	0.5745	0.672	0.5745
1		1.0869	1.1582	0.5745	0.7733	0.5745	0.672	0.5745	0.8847	0.7733	0.8847
1		0.7733	0.672	0.7733	0.672	0.7733	0.672	0.7733	0.5745	0.672	0.5745
1		0.672	0.8847	0.5745	0.672	0.5745	0.7733	0.672	0.7733	0.672	0.7733
1	sponge	0.4795	0.2705	0.5745	0.3722	0.4795	0.3722	0.5745	0.4795	0.5745	0.4795
1		0.672	0.7733	0.4795	0.5745	0.4795	0.5745	0.3722	0.4795	0.3722	0.4795
1		0.4795	0.3722	0.4795	0.3722	0.672	0.3722	0.4795	0.3722	0.4795	0.3722
1		0.3722	0.672	0.4795	0.5745	0.4795	0.5745	0.3722	0.4795	0.3722	0.4795
1	silicon	0.4795	0.3722	0.672	0.5745	0.672	0.4795	0.5745	0.4795	0.5745	0.4795
1		0.672	0.7733	0.672	0.7733	0.672	0.7733	0.5745	0.672	0.5745	0.672
1		0.5745	0.4795	0.672	0.5745	0.7733	0.672	0.7733	0.5745	0.672	0.5745
1		0.4795	0.5745	0.4795	0.5745	0.4795	0.5745	0.4795	0.672	0.5745	0.7733
2	wood	0.4795	0.2705	0.672	0.4795	0.672	0.5745	0.7733	0.672	0.7733	0.672
2		0.5745	0.7733	0.5745	0.7733	0.672	0.7733	0.672	0.7733	0.672	0.7733
2		0.2705	0.1754	0.7733	0.672	0.7733	0.5745	0.7733	0.5745	0.672	0.5745
2		0.672	0.7733	0.672	0.7733	0.672	0.7733	0.672	0.7733	0.672	0.7733
2	sponge	0.2705	0.1754	0.5745	0.3722	0.5745	0.3722	0.5745	0.3722	0.4795	0.3722
2		0.4795	0.5745	0.4795	0.5745	0.3722	0.4795	0.3722	0.5745	0.4795	0.5745
2		0.5745	0.4795	0.5745	0.4795	0.5745	0.3722	0.5745	0.4795	0.672	0.4795
2		0.672	0.8847	0.672	0.7733	0.672	0.7733	0.672	0.7733	0.5745	0.672
2	silicon	0.672	0.5745	0.672	0.5745	0.672	0.4795	0.7733	0.5745	0.672	0.4795
2		0.672	0.7733	0.672	0.7733	0.5745	0.7733	0.672	0.7733	0.672	0.7733
2		0.7733	0.672	0.7733	0.672	0.7733	0.672	0.7733	0.5745	0.672	0.4795
2		0.5745	0.7733	0.5745	0.7733	0.4795	0.7733	0.672	0.7733	0.5745	0.672
3	wood	0.3722	0.2705	0.5745	0.2705	0.7733	0.4795	0.672	0.5745	0.7733	0.672
3		1.1582	1.1582	1.1582	0.9733	1.0869	0.5745	0.672	0.2705	0.4795	0.3722
3		0.1754	0.1754	0.2705	0.1754	0.672	0.5745	0.7733	0.4795	0.5745	0.4795
3		1.1582	1.1582	0.7733	0.8847	0.672	0.8847	0.7733	0.8847	0.5745	0.7733
3	sponge	0.3722	0.2705	0.5745	0.3722	0.4795	0.3722	0.5745	0.4795	0.5745	0.4795
3		0.9733	1.0869	0.7733	0.8847	0.672	0.7733	0.672	0.7733	0.4795	0.5745
3		0.1754	0.1754	0.4795	0.3722	0.672	0.5745	0.7733	0.3722	0.4795	0.3722
3		0.8847	1.0869	0.672	0.7733	0.3722	0.5745	0.4795	0.672	0.5745	0.672
3	silicon	0.5745	0.4795	0.672	0.5745	0.7733	0.672	0.7733	0.672	0.8847	0.672
3		1.0869	1.1582	0.672	0.7733	0.672	0.8847	0.672	0.7733	0.672	0.7733
3		0.4795	0.3722	0.4795	0.3722	0.4795	0.3722	0.4795	0.3722	0.5745	0.4795
3		0.672	0.7733	0.672	0.8847	0.7733	0.8847	0.672	0.7733	0.672	0.7733
4	wood	0.4795	0.3722	0.5745	0.3722	0.5745	0.4795	0.672	0.5745	0.672	0.5745
4		0.672	0.7733	0.672	0.7733	0.672	0.7733	0.672	0.7733	0.4795	0.7733
4		0.5745	0.4795	0.5745	0.4795	0.5745	0.2705	0.672	0.4795	0.672	0.4795
4		0.9733	1.0869	0.8847	0.9733	0.672	0.8847	0.5745	0.7733	0.672	0.7733
4	sponge	0.4795	0.3722	0.4795	0.3722	0.4795	0.3722	0.7733	0.5745	0.672	0.5745
4		0.672	0.7733	0.3722	0.672	0.4795	0.7733	0.672	0.7733	0.672	0.7733
4		0.4795	0.3722	0.5745	0.4795	0.5745	0.4795	0.5745	0.4795	0.672	0.5745
4		0.4795	0.7733	0.5745	0.672	0.4795	0.5745	0.4795	0.5745	0.3722	0.4795

4	silicon	0.4795	0.3722	0.7733	0.672	0.7733	0.4795	0.672	0.5745	0.7733	0.672
4		0.5745	0.7733	0.5745	0.7733	0.672	0.7733	0.672	0.7733	0.5745	0.7733
4		0.4795	0.3722	0.5745	0.4795	0.7733	0.672	0.7733	0.672	0.7733	0.672
4		1.1582	1.1582	0.672	0.7733	0.5745	0.672	0.4795	0.7733	0.672	0.7733
5	wood	0.7733	0.672	0.7733	0.672	0.7733	0.672	0.7733	0.672	0.7733	0.672
5		0.7733	0.8847	0.4795	0.8847	0.672	0.7733	0.672	0.8847	0.672	0.7733
5		0.5745	0.4795	0.672	0.5745	0.672	0.5745	0.7733	0.672	0.7733	0.672
5		0.7733	0.8847	0.5745	0.672	0.4795	0.672	0.3722	0.4795	0.2705	0.7733
5	sponge	0.4795	0.3722	0.4795	0.3722	0.5745	0.4795	0.5745	0.4795	0.672	0.4795
5		0.672	0.7733	0.672	0.7733	0.4795	0.5745	0.3722	0.5745	0.4795	0.7733
5		0.3722	0.2705	0.3722	0.2705	0.4795	0.3722	0.4795	0.3722	0.4795	0.2705
5		0.5745	0.7733	0.672	0.7733	0.4795	0.5745	0.3722	0.4795	0.3722	0.4795
5	silicon	0.5745	0.4795	0.672	0.5745	0.672	0.5745	0.7733	0.5745	0.672	0.5745
5		0.672	0.7733	0.672	0.7733	0.672	0.7733	0.5745	0.672	0.4795	0.5745
5		0.672	0.4795	0.672	0.4795	0.672	0.4795	0.7733	0.5745	0.672	0.5745
5		0.5745	0.7733	0.672	0.7733	0.672	0.7733	0.672	0.7733	0.672	0.7733
6	wood	0.3722	0.2705	0.3722	0.2705	0.8847	0.5745	0.672	0.5745	0.7733	0.672
6		0.5745	0.7733	0.5745	0.672	0.4795	0.5745	0.2705	0.4795	0.2705	0.5745
6		0.4795	0.3722	0.4795	0.1754	0.3722	0.2705	0.3722	0.2705	0.5745	0.2705
6		1.1582	1.1582	0.7733	0.8847	0.4795	0.5745	0.4795	0.672	0.5745	0.7733
6	sponge	0.4795	0.3722	0.5745	0.4795	0.7733	0.672	0.7733	0.672	0.8847	0.672
6		0.8847	0.9733	0.2705	0.7733	0.672	0.7733	0.5745	0.672	0.3722	0.672
6		0.5745	0.2705	0.4795	0.3722	0.672	0.3722	0.4795	0.3722	0.4795	0.2705
6		0.672	0.7733	0.4795	0.7733	0.672	0.7733	0.672	0.8847	0.672	0.7733
6	silicon	0.5745	0.4795	0.5745	0.4795	0.672	0.5745	0.7733	0.672	0.7733	0.5745
6		0.672	0.7733	0.5745	0.7733	0.672	0.7733	0.672	0.7733	0.672	0.7733
6		0.4795	0.3722	0.5745	0.4795	0.672	0.3722	0.7733	0.5745	0.672	0.5745
6		0.672	0.7733	0.5745	0.7733	0.672	0.7733	0.4795	0.5745	0.4795	0.5745
7	wood	0.3722	0.2705	0.7733	0.672	0.7733	0.2705	0.5745	0.4795	0.8847	0.672
7		0.672	0.7733	0.672	0.7733	0.5745	0.672	0.5745	0.8847	0.672	0.7733
7		0.5745	0.4795	0.5745	0.4795	0.8847	0.672	0.9733	0.672	0.7733	0.672
7		0.9733	1.0869	0.8847	0.9733	0.672	0.7733	0.3722	0.4795	0.3722	0.7733
7	sponge	0.7733	0.5745	0.672	0.3722	0.4795	0.2705	0.3722	0.2705	0.3722	0.2705
7		0.7733	0.8847	0.672	0.7733	0.672	0.7733	0.4795	0.5745	0.2705	0.5745
7		0.5745	0.3722	0.4795	0.3722	0.5745	0.4795	0.5745	0.4795	0.5745	0.4795
7		0.672	0.7733	0.5745	0.672	0.5745	0.7733	0.4795	0.5745	0.4795	0.5745
7	silicon	0.3722	0.2705	0.5745	0.4795	0.5745	0.3722	0.5745	0.4795	0.5745	0.4795
7		0.8847	0.9733	0.3722	0.5745	0.4795	0.672	0.3722	0.5745	0.3722	0.7733
7		0.3722	0.2705	0.672	0.4795	0.5745	0.4795	0.8847	0.4795	0.5745	0.4795
7		1.1582	1.1582	0.672	0.7733	0.5745	0.7733	0.672	0.7733	0.5745	0.7733
8	wood	0.5745	0.4795	0.672	0.5745	0.7733	0.4795	0.5745	0.4795	0.7733	0.5745
8		0.5745	0.7733	0.672	0.7733	0.672	0.7733	0.672	0.7733	0.672	0.7733
8		0.7733	0.672	0.7733	0.5745	0.672	0.5745	0.7733	0.5745	0.7733	0.672
8		0.672	0.7733	0.672	0.7733	0.672	0.7733	0.672	0.7733	0.672	0.7733
8	sponge	0.3722	0.2705	0.5745	0.3722	0.4795	0.3722	0.5745	0.3722	0.4795	0.3722
8		0.3722	0.5745	0.4795	0.5745	0.3722	0.4795	0.3722	0.5745	0.4795	0.5745
8		0.2705	0.1754	0.3722	0.2705	0.3722	0.2705	0.3722	0.2705	0.4795	0.3722
8		0.3722	0.4795	0.3722	0.4795	0.3722	0.4795	0.3722	0.5745	0.3722	0.4795

8	silicon	0.5745	0.4795	0.7733	0.5745	0.7733	0.672	0.7733	0.672	0.7733	0.4795
8		0.672	0.7733	0.4795	0.672	0.5745	0.7733	0.672	0.7733	0.672	0.7733
8		0.7733	0.5745	0.672	0.4795	0.7733	0.5745	0.7733	0.5745	0.672	0.5745
8		0.5745	0.7733	0.672	0.7733	0.5745	0.672	0.5745	0.672	0.5745	0.7733
9	wood	0.672	0.5745	0.672	0.2705	0.4795	0.2705	0.3722	0.2705	0.4795	0.3722
9		0.4795	0.672	0.5745	0.8847	0.7733	0.8847	0.5745	0.7733	0.3722	0.7733
9		0.3722	0.2705	0.5745	0.4795	0.672	0.5745	0.672	0.5745	0.672	0.4795
9		0.672	0.7733	0.4795	0.7733	0.5745	0.7733	0.672	0.7733	0.672	0.7733
9	sponge	0.4795	0.3722	0.5745	0.3722	0.5745	0.3722	0.7733	0.672	0.8847	0.672
9		0.8847	0.9733	0.672	0.7733	0.4795	0.672	0.5745	0.7733	0.672	0.7733
9		0.4795	0.2705	0.4795	0.3722	0.4795	0.3722	0.4795	0.2705	0.5745	0.4795
9		0.672	0.7733	0.3722	0.4795	0.3722	0.672	0.5745	0.7733	0.672	0.7733
9	silicon	0.4795	0.3722	0.5745	0.3722	0.5745	0.4795	0.672	0.5745	0.7733	0.5745
9		0.672	0.7733	0.5745	0.7733	0.672	0.7733	0.672	0.7733	0.4795	0.672
9		0.4795	0.3722	0.672	0.5745	0.672	0.5745	0.7733	0.4795	0.5745	0.4795
9		0.5745	0.672	0.4795	0.5745	0.4795	0.5745	0.4795	0.672	0.5745	0.672
10	wood	0.672	0.4795	0.5745	0.3722	0.5745	0.4795	0.672	0.5745	0.7733	0.672
10		0.672	0.7733	0.2705	0.5745	0.4795	0.672	0.4795	0.672	0.5745	0.7733
10		0.5745	0.3722	0.4795	0.3722	0.7733	0.672	0.7733	0.672	0.7733	0.672
10		0.8847	0.9733	0.672	0.8847	0.5745	0.7733	0.672	0.7733	0.672	0.7733
10	sponge	0.5745	0.4795	0.5745	0.4795	0.5745	0.3722	0.672	0.4795	0.672	0.2705
10		0.3722	0.4795	0.3722	0.4795	0.2705	0.4795	0.3722	0.5745	0.4795	0.7733
10		0.4795	0.3722	0.5745	0.4795	0.5745	0.4795	0.5745	0.4795	0.672	0.3722
10		1.1582	1.1582	0.4795	0.5745	0.3722	0.5745	0.4795	0.5745	0.4795	0.7733
10	silicon	0.5745	0.3722	0.4795	0.2705	0.3722	0.2705	0.5745	0.2705	0.7733	0.4795
10		0.8847	0.9733	0.4795	0.5745	0.4795	0.5745	0.4795	0.5745	0.4795	0.5745
10		0.1754	0.1754	0.7733	0.672	0.7733	0.4795	0.5745	0.4795	0.5745	0.4795
10		1.1582	1.1582	0.672	0.7733	0.672	0.7733	0.672	0.7733	0.672	0.7733

Experiment 2

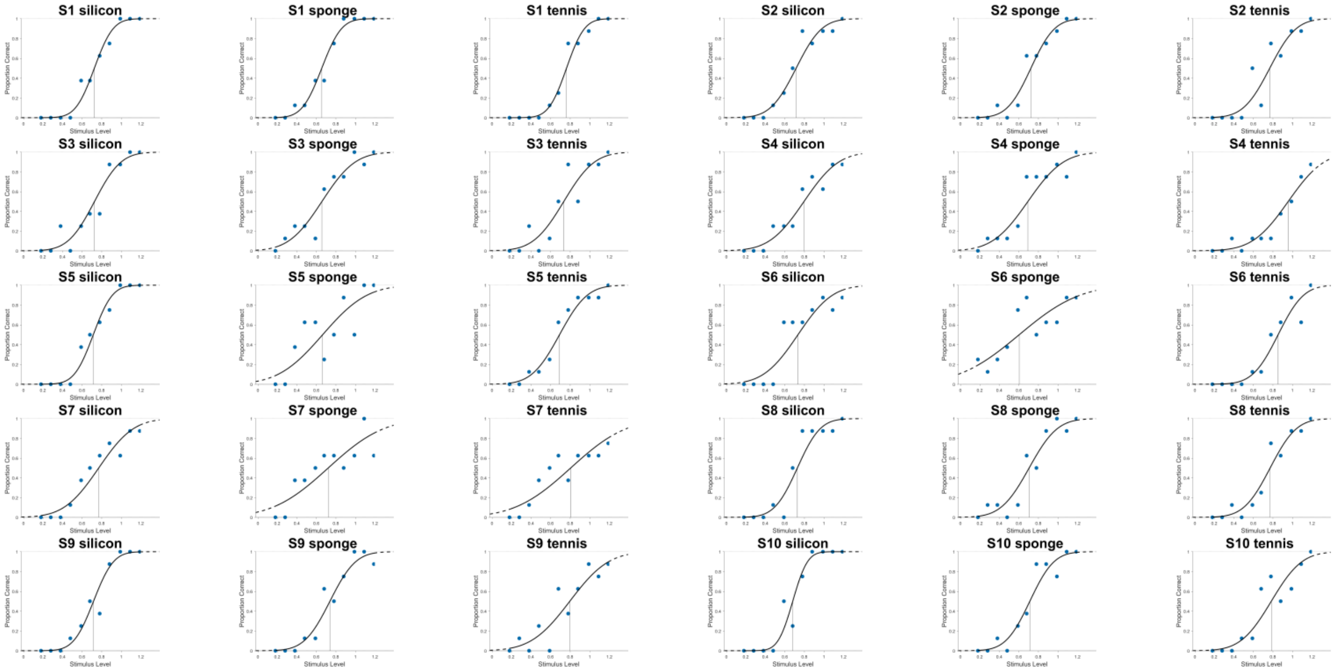

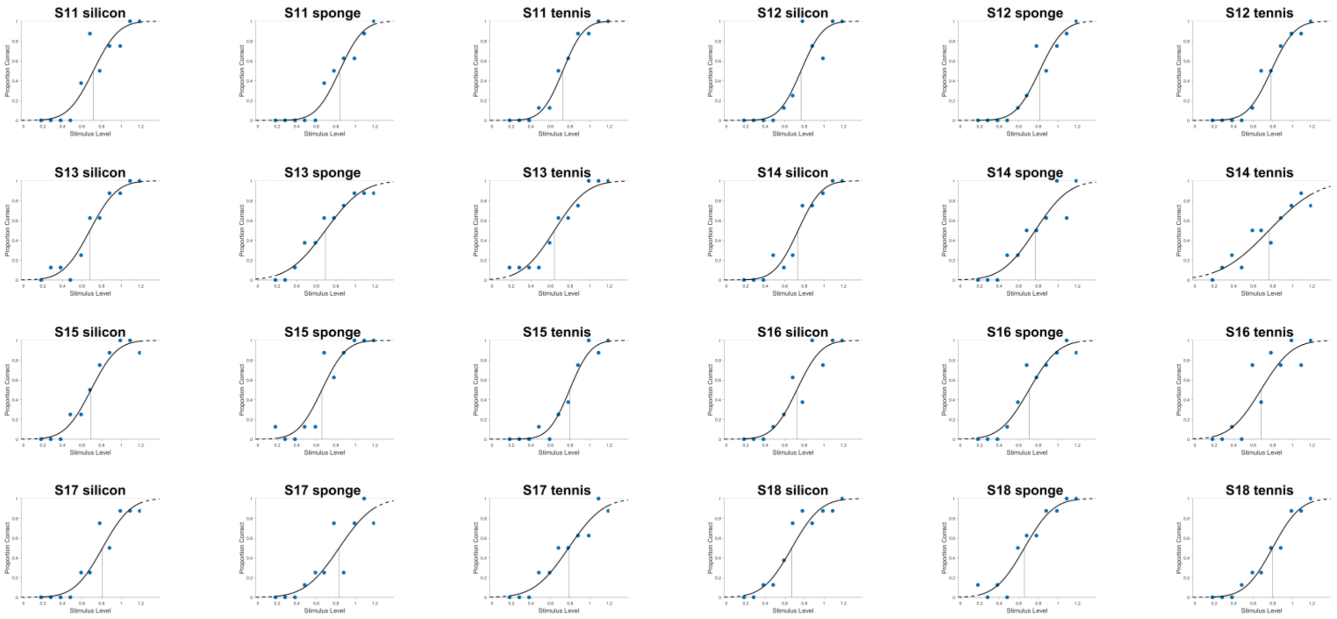

Experiment 3

Subject Nr	*Condition*	*Matches in mm/N*							
1	foam ball	0.7733	0.672	0.8847	0.8847	0.9733	0.7733	0.7733	0.8847
1	tennis ball	0.3722	0.4795	0.7733	0.4795	0.9733	0.7733	0.7733	0.672
1	sponge	0.3722	0.5745	0.9733	0.9733	0.8847	1.0869	1.0869	0.7733
1	silicon	0.7733	0.7733	0.9733	0.7733	0.672	1.0869	0.8847	0.8847
2	foam ball	0.4795	0.4795	0.5745	0.4795	0.4795	0.5745	0.7733	0.672
2	tennis ball	0.4795	0.3722	0.4795	0.672	0.4795	0.5745	0.4795	0.672
2	sponge	0.4795	0.4795	0.672	0.5745	0.4795	0.4795	0.5745	0.4795
2	silicon	0.3722	0.4795	0.4795	0.5745	0.5745	0.4795	0.672	0.672
3	foam ball	0.9733	0.7733	0.672	0.7733	0.672	0.7733	0.4795	0.7733
3	tennis ball	0.5745	0.7733	0.672	0.4795	0.5745	0.672	0.672	0.4795
3	sponge	0.5745	0.7733	0.672	0.672	0.7733	0.672	0.672	0.5745
3	silicon	0.672	0.7733	0.5745	0.7733	0.5745	0.672	0.4795	0.7733
4	foam ball	0.8847	0.4795	0.4795	0.672	0.4795	0.672	0.4795	0.8847
4	tennis ball	0.4795	0.7733	0.4795	0.5745	0.3722	0.672	0.3722	0.7733
4	sponge	0.5745	0.7733	0.672	0.3722	0.3722	0.7733	0.8847	0.3722
4	silicon	0.7733	0.5745	0.4795	0.672	0.3722	0.7733	0.4795	0.4795
5	foam ball	0.7733	0.7733	0.672	0.5745	0.5745	0.5745	0.4795	0.672
5	tennis ball	0.4795	0.7733	0.5745	0.672	0.3722	0.4795	0.4795	0.4795
5	sponge	0.7733	0.5745	0.7733	0.8847	0.672	0.7733	0.672	0.7733
5	silicon	0.672	0.4795	0.4795	0.4795	0.3722	0.4795	0.3722	0.4795
6	foam ball	0.672	0.4795	0.5745	0.4795	0.672	0.4795	0.672	0.3722
6	tennis ball	0.3722	0.3722	0.4795	0.4795	0.3722	0.3722	0.4795	0.4795
6	sponge	0.3722	0.5745	0.3722	0.5745	0.4795	0.3722	0.3722	0.4795
6	silicon	0.4795	0.5745	0.5745	0.3722	0.4795	0.4795	0.672	0.4795
7	foam ball	0.5745	0.4795	0.7733	0.672	0.7733	0.5745	0.672	0.8847
7	tennis ball	0.4795	0.4795	0.672	0.5745	0.5745	0.5745	0.672	0.7733
7	sponge	0.672	0.4795	0.5745	0.7733	0.672	0.5745	0.7733	0.7733
7	silicon	0.5745	0.4795	0.4795	0.672	0.7733	0.4795	0.5745	0.5745

8	foam ball	0.5745	0.672	0.7733	0.5745	0.672	0.4795	0.8847	0.4795
8	tennis ball	0.4795	0.672	0.672	0.4795	0.672	0.4795	0.4795	0.7733
8	sponge	0.672	0.4795	0.7733	0.4795	0.672	0.4795	0.4795	0.672
8	slilicon	0.4795	0.5745	0.4795	0.7733	0.4795	0.672	0.4795	0.672
9	foam ball	0.672	0.672	0.7733	0.5745	0.8847	0.5745	0.5745	0.672
9	tennis ball	0.4795	0.5745	0.7733	0.5745	0.5745	0.7733	0.4795	0.4795
9	sponge	0.672	0.672	0.672	0.7733	0.5745	0.672	0.672	0.672
9	slilicon	0.4795	0.7733	0.672	0.672	0.672	0.7733	0.5745	0.4795
10	foam ball	0.7733	0.7733	0.8847	0.8847	0.8847	0.8847	0.9733	0.5745
10	tennis ball	0.8847	0.8847	0.8847	0.8847	0.8847	0.672	0.5745	0.7733
10	sponge	0.8847	0.8847	0.8847	0.7733	0.672	0.7733	0.5745	0.7733
10	slilicon	0.8847	0.9733	0.8847	1.0869	0.7733	0.7733	0.7733	0.5745
11	foam ball	0.4795	0.5745	0.3722	0.4795	0.4795	0.7733	0.672	0.4795
11	tennis ball	0.4795	0.5745	0.4795	0.4795	0.7733	0.4795	0.3722	0.4795
11	sponge	0.3722	0.5745	0.4795	0.4795	0.4795	0.4795	0.3722	0.3722
11	slilicon	0.7733	0.4795	0.5745	0.4795	0.5745	0.4795	0.5745	0.7733
12	foam ball	0.7733	0.4795	0.4795	0.5745	0.5745	0.8847	0.4795	0.7733
12	tennis ball	0.672	0.5745	0.5745	0.3722	0.4795	0.672	0.4795	0.672
12	sponge	0.5745	0.4795	0.4795	0.7733	0.4795	0.7733	0.5745	0.7733
12	slilicon	0.4795	0.672	0.7733	0.7733	0.7733	0.7733	0.5745	0.7733
13	foam ball	0.4795	0.3722	0.4795	0.4795	0.4795	0.4795	0.4795	0.4795
13	tennis ball	0.3722	0.3722	0.4795	0.4795	0.3722	0.3722	0.4795	0.3722
13	sponge	0.3722	0.4795	0.4795	0.3722	0.4795	0.3722	0.4795	0.3722
13	slilicon	0.4795	0.3722	0.3722	0.4795	0.3722	0.3722	0.4795	0.3722
14	foam ball	0.7733	0.5745	0.7733	0.5745	0.7733	0.4795	0.7733	0.5745
14	tennis ball	0.4795	0.7733	0.7733	0.4795	0.7733	0.672	0.672	0.5745
14	sponge	0.7733	0.672	0.7733	0.5745	0.672	0.4795	0.5745	0.4795
14	slilicon	0.4795	0.8847	0.5745	0.672	0.7733	0.5745	0.5745	0.4795

Experiment 4

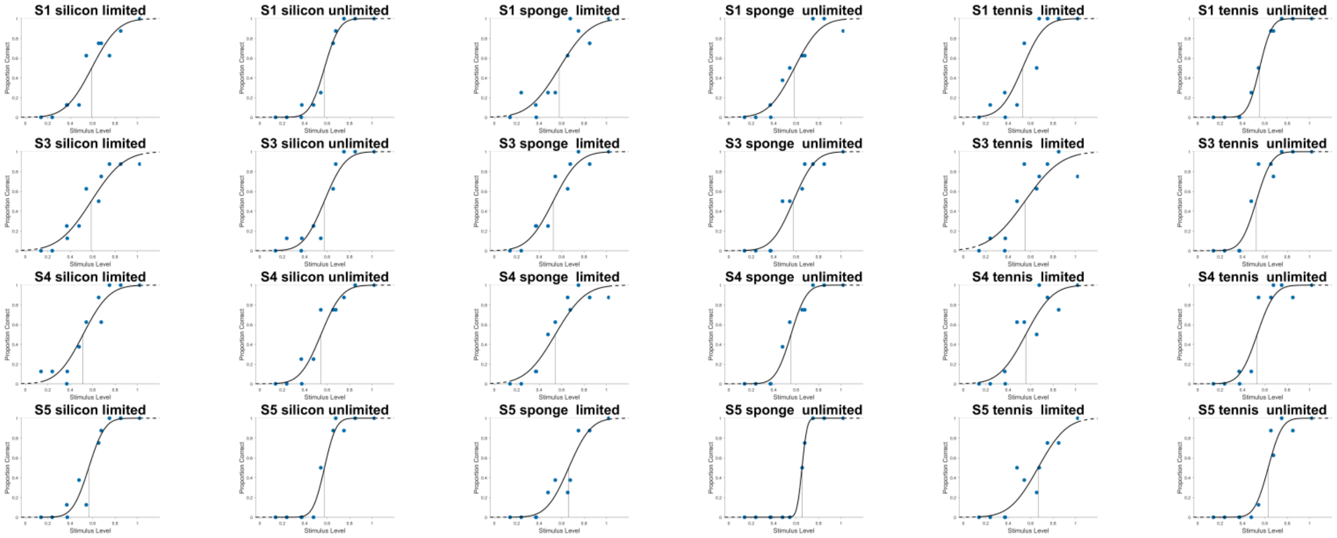

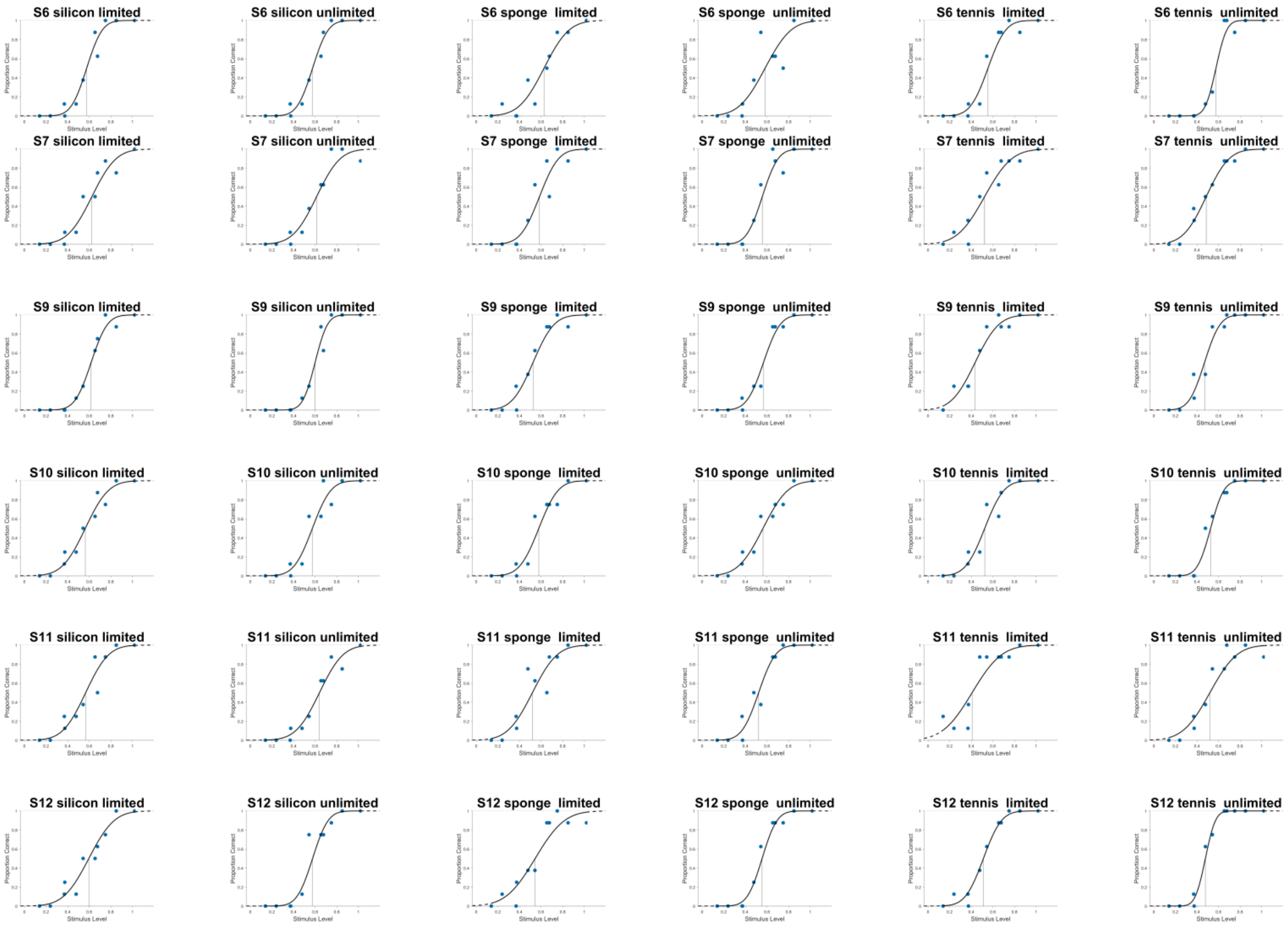
S6 silicon limited
S6 silicon unlimited
S6 sponge limited
S6 sponge unlimited
S6 tennis limited
S6 tennis unlimited
S7 silicon limited
S7 silicon unlimited
S7 sponge limited
S7 sponge unlimited
S7 tennis limited
S7 tennis unlimited
S9 silicon limited
S9 silicon unlimited
S9 sponge limited
S9 sponge unlimited
S9 tennis limited
S9 tennis unlimited
S10 silicon limited
S10 silicon unlimited
S10 sponge limited
S10 sponge unlimited
S10 tennis limited
S10 tennis unlimited
S11 silicon limited
S11 silicon unlimited
S11 sponge limited
S11 sponge unlimited
S11 tennis limited
S11 tennis unlimited
S12 silicon limited
S12 silicon unlimited
S12 sponge limited
S12 sponge unlimited
S12 tennis limited
S12 tennis unlimited
